# Population biology of 225 serogroup 6 *Streptococcus pneumoniae* isolates collected in China

**DOI:** 10.1186/1471-2334-14-467

**Published:** 2014-08-27

**Authors:** Wei Shi, Kaihu Yao, Mingming He, Sangjie Yu, Yonghong Yang

**Affiliations:** Key Laboratory of Major Diseases in Children and National Key Discipline of Pediatrics (Capital Medical University), Ministry of Education, Beijing Pediatric Research Institute, Beijing Children’s Hospital Capital Medical University, Beijing, China

**Keywords:** *Streptococcus pneumoniae*, Serotypes, Antibiotic resistance, Children, Epidemiology

## Abstract

**Background:**

In this study, we defined the population biology of serogroup 6 *Streptococcus pneumoniae* collected in China and their antibiotic resistance profiles.

**Methods:**

The serotypes of 225 *S. pneumoniae* strains isolated between 1997 and 2011 were identified with the Quellung reaction and serotype-specific PCR. All isolated pneumococci were tested for their sensitivity to 11 kinds of antibiotics with the E-test method or disc diffusion. The sequence types (STs) were analyzed with multilocus sequence typing (MLST).

**Results:**

The frequencies of serotypes and subtypes 6A, 6B-I, 6B-II, 6C, and 6D among the 225 isolates were 46.7% (105/225), 19.6% (44/225), 25.8% (58/225), 6.2% (14/225), and 1.8% (4/225), respectively. Serotype 6E was not found in the serotype 6A isolates, and neither serotype 6F nor 6G was identified in any isolate. MLST analysis revealed 58 STs. The most common STs were ST982 (23.1%), ST90 (14.7%), ST4542 (7.6%), and ST2912 (4.9%). The rates of clonal complex 90 (CC90) and CC386 among the oral-penicillin-nonsusceptible isolates decreased over the years, whereas the rates of CC855 and CC3173 increased. The four CCs had similar penicillin MIC distributions, with a maximum MIC of 2 μg/ml.

**Conclusions:**

This study identified the serotypes/subtypes and CCs/STs of group 6 *S. pneumoniae* present in China. No salient antibiotic-resistant clones were isolated among the serogroup 6 *S. pneumoniae*.

**Electronic supplementary material:**

The online version of this article (doi:10.1186/1471-2334-14-467) contains supplementary material, which is available to authorized users.

## Background

*Streptococcus pneumoniae* remains the leading cause of community-acquired respiratory infections, and is one of the most important human pathogens causing severe invasive infections [1]. So far, more than 90 pneumococcal serotypes, classified into 46 groups, have been identified. Serogroup 6 is one of the most common causes of invasive pneumococcal disease in unvaccinated populations. Serogroup 6 traditionally comprises two serotypes, 6A and 6B. Two new serotypes, 6C and 6D, previously identified as serotype 6A and 6B, respectively, by the Quellung reaction were identified as two distinct serotypes using a multibead assay with monoclonal antibodies [2, 3]. A factor antiserum, 6d, was then developed against these two new serotypes. Therefore, these two serotypes can now be identified with the traditional Quellung test [4, 5]. Two subtypes of 6B, designated 6B-I and 6B-II, can be distinguished by polymerase chain reaction (PCR) [6]. Another study in Korea demonstrated that some serogroup 6 isolates encode distinctive *cps* (capsular) loci, differing from those of most other serogroup 6 isolates. These were designated the “6X” group, and include both traditional serotype 6A and 6B isolates [7]. Based on the differences in their capsular gene sequences, serotype “6E” was assigned to this group in a subsequent study [8]. In a German epidemiological survey, two serogroup 6 isolates could not be assigned to a serotype using factor sera [9]. The latest report by Oliver et al. [10] showed that the two isolates were assigned to new serotypes, “6F” and “6G”. That study showed that a single codon change in the *cps* gene, encoding a glycosyl transferase, altered the substrate specificity, caused the biosynthesis of heterogeneous capsule repeating units, and resulted in a new hybrid capsule type. Antigenic formulae and specific factor antisera against serotypes “6E”, “6F”, and “6G” have not yet been developed, but are necessary for their traditional serotype classification [11]. Because the new serotypes have not been confirmed with the traditional Quellung test, they are designated with quotation marks in the present text.

We did not want to investigate the effects of the vaccine PCV7, because its immunization coverage is too low (<1%) to reflect its effectiveness. We set out to identify any new serotypes (serotypes “6E”, “6F”, and “6G”) in serogroup 6 *S. pneumoniae* in China and to report the population biology of this group. In our previous study [12], we examined the genetic structures of only serotypes 6C and 6D, without the most frequent serotypes 6A and 6B. In the present study, we report the STs of all the serogroup 6 isolates and an association between the STs and antibiotic resistance. We intended to use identical isolates in these two studies, but some isolates from our previous study were dead when we went to include them in the present study. Therefore, the present study includes some new isolates collected in 2008–2011 from Shenzhen and Beijing. There were differences in the antibiotic resistance in different clones. For completeness, some published data or similar information from our previous paper have been included in the present results, but these are not included in the analysis or discussion.

## Methods

### Pneumococcal isolates

A total of 225 serogroup 6 *S. pneumoniae* strains isolated from children in 1997–2011 were included in the study. The details of these *S. pneumoniae* collections have been published previously [13–18]. The isolates were cultured from nasopharyngeal specimens, except those collected from Beijing in 2006–2008 and from Shenzhen in 2009–2011, which were cultured from hypopharyngeal aspirate specimens. All isolates were identified as serotypes 6A, 6B, 6C, and 6D with a capsule Quellung test using a pneumotest kit and specific factor antisera (Statens Serum Institut, Copenhagen, Denmark). Typing was performed with phase-contrast microscopy, according to previously reported procedures [19].

A parent and/or legal guardian of each participant signed a written informed consent document before enrollment and before any study procedure was performed. This study was viewed and approved by the Ethics Committee of Beijing Children’s Hospital Affiliated to Capital Medical University. No ethical problems existed in this study.

### PCR identification of 6A, 6B-I, 6B-II, 6C, 6D, “6E”, “6F”, and “6G”

Chromosomal DNA was extracted from overnight cultures of *S. pneumoniae* isolates grown on 5% trypticase soy agar (Oxoid Ltd, Basingstoke, England) using the SiMax™ Genomic DNA Extraction Kit (SBS Genetech Co., Ltd), according to the manufacturer’s instructions. The 6C isolates were differentiated from the 6A and “6E” isolates using PCR with primers 5106 and 3101, as described previously [6, 7, 20], which generated a 1.8-kilobase-pair (kbp) amplicon for 6C and a 2.0-kbp or 2.3-kbp amplicon for 6A or “6E”, respectively. This pair of primers was also used to differentiate the 6D *cps* locus from that of 6B, generating a 1.8-kbp amplicon for 6D and a 2.0-kbp or 2.3-kbp amplicon for subtypes 6B-I or 6B-II, respectively. The primers 5101 and 3101 used to differentiate 6A and “6E”, as well as 6B-I and 6B-II, more definitively generated a 956-bp amplicon for 6A/6B-I and a 1.2-kbp amplicon for “6E”/6B-II [6, 7]. The PCR procedures were as previously described [20]. All serotype 6A and 6B isolates were retested against the factor antiserum 6d and all serotype 6C and 6D isolates were retested against factor 6b or 6c to identify serotype “6 F” or “6G”, respectively, which could not be assigned to serotype 6A or 6C and 6B or 6D, respectively. All isolates that reacted with factor 6d were subjected to PCR amplification for *wciP* locus with primers 5106/3101 and *wciN* locus with primers 5108/3107, as reported previously, to identify serotypes “6 F” and “6G”, respectively [21].

### Antimicrobial susceptibility

The minimum inhibitory concentrations (MICs) of all isolates were determined for penicillin, amoxicillin–clavulanic acid, ceftriaxone, cefuroxime, erythromycin, imipenem, levofloxacin, and vancomycin using E-test strips (AB Biodisk, Solna, Sweden), and their susceptibility to tetracycline, sulfamethoxazole–trimethoprim, and chloramphenicol was assessed with disc diffusion tests (Oxoid). The use of the penicillin parenteral (nonmeningitis) breakpoints for susceptible, intermediate, and resistant rates to compare penicillin susceptibilities in this study blurred the differences between the serotypes [22]. Therefore, the oral penicillin V breakpoints were also used to analyze penicillin susceptibility. *Streptococcus pneumoniae* ATCC49619 was used as the reference strain in the susceptibility tests. Isolates were considered multidrug resistant if they were not susceptible to three or more classes of antimicrobials.

### Molecular epidemiology of serogroup 6 strains

All strains were characterized with multilocus sequence typing (MLST). Bacterial chromosomal DNA was extracted as described above. The PCR fragments of the seven MLST genes (*aroE*, *gdh*, *gki*, *recP*, *spi*, *xpt*, and *ddl*) were amplified from the chromosomal DNA. The products were sent to BGI Company (Beijing, China) for sequencing on both strands. The STs were determined by comparing the allelic profiles with the recognized STs at the MLST website (http://spneumoniae.mlst.net). The new alleles and allelic profiles identified in the present study have been submitted to the MLST database for name assignment. The eBURST v3 software (available at http://www.mlst.net) was used to estimate the relationships among the isolates and to assign strains to a clonal complex (CC) using the stringent group definition of six of seven shared alleles [23].

### Statistical analysis

The serotyping and antimicrobial resistance data were analyzed with the WHONET 5.3 software, as recommended by the World Health Organization (http://www.who.int/drugresistance/whonetsoftware/en/). The *χ*^2^ test, performed with the SPSS software v. 13.0 (SPSS Inc. USA), was used for statistical comparisons. A two-tailed cut-off of P < 0.05 was deemed to indicate statistical significance.

## Results

### Serotype/subtype distributions and antibiotic susceptibility

Among the 225 isolates identified as serogroup 6 *S. pneumoniae*, the rates of serotypes and subtypes 6A, 6B-I, 6B-II, 6C, and 6D were 46.7% (105/225), 19.6% (44/225), 25.8% (58/225), 6.2% (14/225), and 1.8% (4/225), respectively. PCR analysis of the serotype 6A isolates with primers 5106/3101 or 5101/3101 always generated the same amplicons as the PCR analysis of 6B-I, indicating that no serotype “6E” was present in the serotype 6A isolates. No isolate was assigned ambiguously to 6A or 6C or to 6B or 6D. The 14 serotype 6C isolates had identical *wciP* and *wciN* sequences. The four serotype 6D isolates also had identical *wciP* and *wciN* sequences. The length of *wciP* was the same as that of the reference sequence CR931638, with 10 random mutations. All *wciN* sequences were 1125 bp long and were identical to the published 6C sequence (JF911510), which is longer than that of CR931638 (945 bp). No *wciN* sequence in the present study suggested serotype “6F” or “6G”.

The analysis of antibiotic resistance revealed similar results to those in our previous report [12], which are shown in the tables with no further description.

### MLST

**Figure 1:**
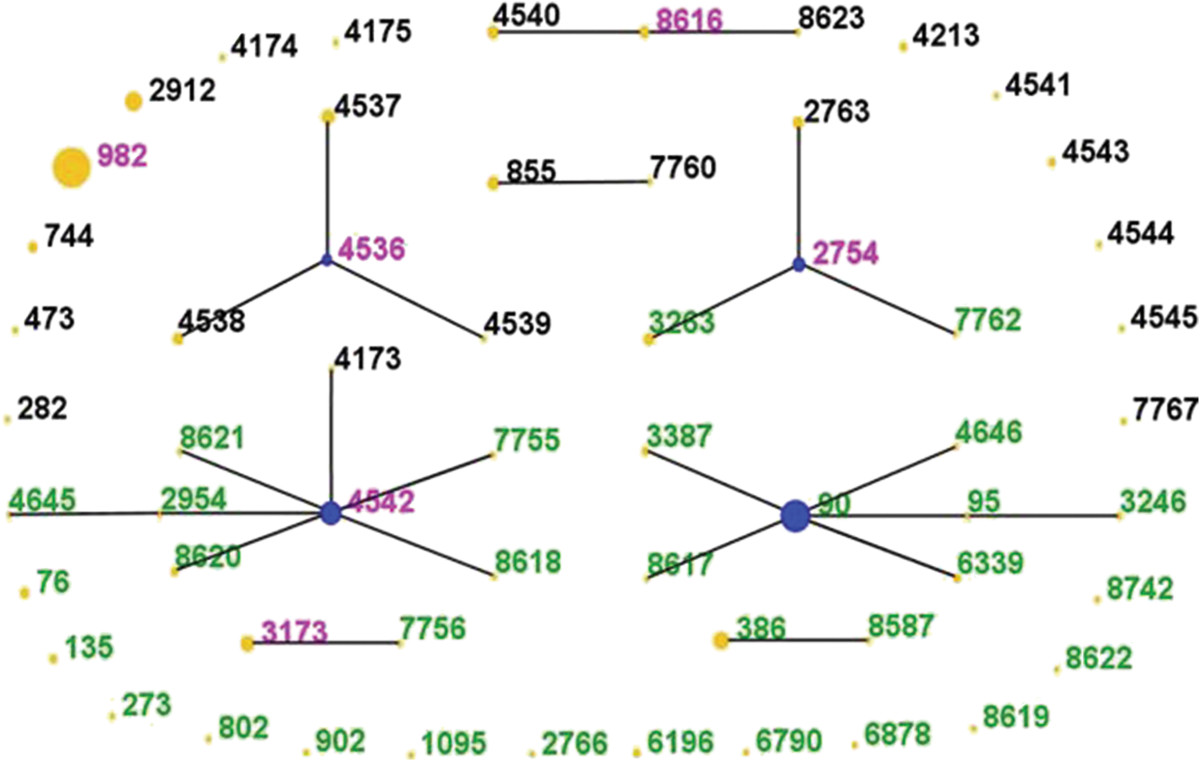
**Population snapshot of 225** ***S. pneumoniae*** **strains revealed by an eBURST analysis.** Lines indicate the presence of single-locus-variant links in particular sequence types, indicated by circles. The size of the circle corresponds to the number of isolates belonging to the sequence type (ST). The green ST numbers indicate serotype 6A or 6C isolates, the black numbers indicate 6B or 6D isolates, and the pink numbers indicate isolates from the two groups.

The MLST analysis revealed 58 STs, the most common of which were ST982 (52 isolates, 23.1%), ST90 (33, 14.7%), ST4542 (17, 7.6%), and ST2912 (11, 4.9%). In this study, we identified eight new STs, ST8617–ST8623 and ST8742, and a novel *gki* locus (*gki*383 in ST8742). An eBURST analysis identified eight CCs and 26 singletons (Figure 1). Two of the common STs, ST982 and ST2912, were singletons. Six CCs each included 10 or more isolates: CC90 (41 isolates), CC4542 (25 isolates), CC4536 (15 isolates), CC2754 (15 isolates), CC386 (10 isolates), and CC8616 (10 isolates). ST982, ST2912, and these six CCs accounted for 79.6% of all the isolates tested.

The distribution of the CCs/STs of the serogroup 6 *S. pneumoniae* isolates according to serotype/subtype is shown in Table 1 and Figure 1. Each serotype/subtype had a main CC/ST that included the majority of the isolates in the serotype/subtype. For instance, the proportion of ST982 in the serotype 6A isolates (44.8%) was much higher than that of any other CC/ST (≤13.3%). Some CCs/STs included several isolates belonging to different serotypes. Most of the ST982 isolates were identified as serotype 6A (47/52); and the other five isolates were serotypes 6C (1/52) and 6D (4/52). Thus, all four 6D isolates were ST982. Serotypes 6A and 6B were both detected in the following STs: ST4542 (6A: 3/17; 6B: 14/17), ST2754 (6A: 2/6, 6B: 4/6), ST3173 (6A: 6/7, 6B: 1/7), ST4536 (6A: 3/4, 6B: 1/4), and ST8616 (6A: 3/4, 6B: 1/4). Other STs could only be detected in one serotype.

**Table 1:** **Distributions of CCs/STs of serogroup 6** ***S. pneumoniae*** **by serotype/subtype**

Types	No.	ST982	CC90	CC4542	CC4536	CC2754	ST2912	CC8616	CC386	Others
6A	105	47(44.8%)	0	3(2.9%)	14(13.3%)	6(5.7%)	2(1.9%)	9(8.6%)	0	24(22.9%)
6B-I	44	0	0	20(46.5%)	1(2.3%)	9(20.9%)	0	1(2.3%)	0	12(27.9%)
6B-II	58	0	41(69.5%)	1(1.7%)	0	0	0	0	10(16.9%)	7(11.9%)
6C	14	1(7.1%)	0	1(7.1%)	0	0	9(64.3%)	0	0	3(21.4%)
6D	4	4(100%)	0	0	0	0	0	0	0	0
Total	225	52(23.1%)	41(18.2%)	25(11.1%)	15(6.7%)	15(6.7%)	11(4.9%)	10(4.4%)	10(4.4%)	46(20.4%)

Note: %, number of serotypes/subtypes divided by number of CCs/STs.

The CC/ST distributions of the serogroup 6 *S. pneumoniae* changed significantly during the study period. The common CCs/STs, such as ST982, CC90, CC4542, and CC4536, mainly occurred in 1997–2005, but these isolates decreased or even disappeared in later years. Two CCs among the common CCs/STs, CC2754 and CC386, were mainly found in 2006–2010, with some fluctuations. No CC or ST showed a clearly increasing trend, except the group involving the infrequent “other” CCs/STs (“others” in Table 1). Considerable isolates identified in this study belonged to the infrequent “other” CCs/STs (46/225), especially in 2009–2011 (10/26).

### Associations between STs/CCs and antimicrobial resistance

The numbers and percentages of isolates not susceptible to oral penicillin and other antibiotics are summarized according to CCs/STs in Tables 2 and 3. Notably, all CC90 isolates were not susceptible to oral penicillin, with high MIC_50_ and MIC_90_ values, whereas other common CCs/STs were usually susceptible, except that some intermediately susceptible strains (40.0%) were present in CC386. More than half the infrequent “other” CC/ST isolates were not susceptible to oral penicillin. High rates of nonsusceptibility to cefuroxime were found in CC90 (92.7%) and the infrequent “other” CCs/STs (45.6%). The differences in some other antimicrobial resistances between the CCs/STs are shown in Table 3.

**Table 2:** **Penicillin susceptibility in serogroup 6** ***S. pneumoniae*** **by CCs/STs**

CCs/STs	No. isolates	Oral penicillin breakpoints		MIC50 (μg/mL)	MIC90 (μg/mL)	MIC range (μg/mL)
		I	R			
ST982	52	0	0	0.032	0.049	0.001–0.064
CC90	41	36(87.8%)	5(12.2%)	1	1.5	0.25–2
CC4542	25	0	0	0.016	0.023	0.003–0.032
ST2912	11	0	0	0.032	0.049	0.016–0.049
CC4536	15	0	0	0.016	0.032	0.016–0.064
CC2754	11	0	0	0.023	0.032	0.008–0.064
CC8616	10	0	0	0.016	0.023	0.002–0.023
CC386	10	4(40.0%)	0	0.064	0.125	0.032–0.125
Others	46	22(47.8%)	4(8.7%)	0.19	0.75	0.008–2
Total	225	63(28.0%)	9(4%)	0.032	1	0.001–2

**Table 3:** **Resistance of serogroup 6** ***S. pneumoniae*** **to several antimicrobials by CCs/STs**

CC/STs	No.	CXM^a^		IPM^a^*	ERY^a^*	CHL^a^*	TCY^a^		SXT^a^		MDR^a^(%)
		I%	R%	I%	R%	R%	I%	R%	I%	R%	
ST982	52	0	0	0	98.1	3.8	1.9	98.1	1.9	98.1	98.1
CC90	41	4.9	87.8	19.5	100	58.5	0	95.1	4.9	92.7	100
CC4542	25	0	0	8.0	96.0	8.0	0	100	4.0	88.0	92.0
ST2912	11	0	0	0	100	0	36.4	45.5	9.1	63.6	63.6
CC4536	15	6.7	0	0	66.7	46.7	0	100	20.0	46.7	73.3
CC2754	15	0	6.7	0	100	6.7	0	93.3	13.3	86.7	93.3
CC8616	10	0	0	10.0	90.0	10.0	0	90.0	0	90.0	90.0
CC386	10	0	0	0	100	0	10.0	80.0	0	10.0	40.0
Others	46	6.5	39.1	8.7	93.5	26.1	4.3	80.4	15.2	63.0	91.3
Total	225	2.7	24.4	6.7	95.1	21.8	3.6	90.2	7.6	78.7	89.8

Note: All 225 strains were susceptible to amoxicillin–clavulanic acid, ceftriaxone, vancomycin, and levofloxacin.

^a^CXM: cefuroxime; IPM: imipenem; ERY: erythromycin; CHL: chloramphenicol; TCY: tetracycline; SXT: sulfamethoxazole; MDR: multidrug resistance.

*No intermediate or resistant isolate was identified.

The distributions of serotypes/subtypes and CCs/STs in the oral-penicillin-nonsusceptible strains (OPNSP) were analyzed further, as shown in Table 4. Although 6B-II was the main subtype in the total OPNSP strains, its proportion decreased from 82.1% in 2000–2002 to 41.7% in 2009–2011 (*χ*^2^ = 5.6, *P* < 0.05). The proportion of serotype 6A strains increased significantly from 10.3% to 50.0% during the same period (*χ*^2^ = 6.8, *P* < 0.01). The constitution of CCs/STs among the OPNSP also changed. The proportions of CC90 and CC386 that were identified as subtype 6B-II decreased. However, the proportions of CC855 (including six serotype 6A isolates) increased, as did the proportions of CC3173 (including six serotype 6A isolates and two subtype 6B-I isolates). The distribution of penicillin MICs between the CCs/STs did not differ, except that the MICs of the CC386 isolates reached 0.125 μg/ml.

**Table 4:** **Distributions of serotypes/subtypes in oral-penicillin-nonsusceptible isolates in China in 2000–2011**

Years	No.	Serotypes/subtypes			CCs/STs				
		6A	6B-I	6B-II	CC90	CC386	CC855	CC3173	Singletons*
2000-2002	39	4(10.3%)	3(7.7%)	32(82.1%)	27(69.2%)	3(7.7%)	2(5.1%)	0(0.0%)	ST76 (3), ST473,ST802, ST902, ST4545
2003-2005	9	2(22.2%)	0(0.0%)	7(77.8%)	6(66.7%)	0(0.0%)	0(0.0%)	1(11.1%)	ST282,ST8742
2006-2008	12	3(25.0%)	2(16.7%)	7(58.3%)	6(50.0%)	0(0.0%)	2(16.7)	2(16.7%)	ST135
2009-2011	12	6(50.0%)	1(8.3%)	5(41.7%)	2(16.7%)	1(8.3%)	2(16.7%)	5(41.7%)	ST135,ST2766
Total	71	15(21.1%)	6(8.5%)	51(71.8%)	41(57.7%)	4(5.6%)	6(8.5%)	8(11.3%)	

Note: No oral-penicillin-nonsusceptible strain was present in 1997–1999.

*The number of isolates is given in parentheses if more than one isolate was identified.

## Discussion

Recently, Ko et al. [8] identified a distinct group among serotypes 6A and 6B *S. pneumoniae* isolates, designated serotype “6E”. Based on sequence data, the serotype “6E” isolates from traditional serotype 6B were the same as those from subtype 6B-II in the present study, which is exactly consistent with subtype 6B-III reported by Elberst et al. [24]. Subtype 6B-II was isolated frequently in the present study. However, no serotype “6E” isolate was identified in traditional serotype 6A with PCR.

As-yet-unconfirmed serotype “6E” has only been identified based on its capsular gene sequence [8] because no new serotype including both traditional serotypes 6A and 6B can be constructed within the serological typing system [11]. If differences in gene sequences indicate new serotypes, two new serotypes must be individually recognized within the traditional serotypes 6A and 6B.

All the common CCs/STs included more than one serotype or subtype, except CC90 and CC386, which included subtype 6B-II isolates only. One subtype 6B-II isolate belonged to CC4542, which otherwise included only serotype 6A and subtype 6B-I isolates. However, this isolate had a unique ST, ST8618. Two classes of *cps* sequences have been reported for serogroup 6 isolates. The class 2 *cps* sequences are limited to a few serotype 6B isolates [25], which are in fact subtype 6B-II. The present data show that our subtype 6B-II isolates displayed genetic backgrounds different from those of other serotypes/subtypes.

Our previous studies have suggested that a clonal shift occurred in serotype 19F and 23F isolates between 1997 and 2010. In the serotype 19F *S. pneumoniae* isolates, the spread of CC271 with high β-lactam-antibiotic resistance may be driven by the selective pressure applied with the overuse of antibiotics, whereas the spread of susceptible CC983 is limited. CC271 became the main population of serotype 19F *S. pneumoniae* after 2003 [26]. In the serotype 23F *S. pneumoniae* isolates, ST81 (with high β-lactam-antibiotic resistance) took the place of ST342 and became the absolutely predominant population in 2010 [27]. Both CC271 and ST81 are also important international epidemic clones [26, 27]. We noted that selection by antibiotic abuse caused an increase in subtype 6B-II isolates with significantly higher β-lactam-antibiotic resistance than that of other serogroup 6 *S. pneumoniae* isolates in 1997–2008 [12]. The present data show that most subtype 6B-II isolates belong to CC90. ST90 is one of the globally epidemic resistant clones, Spain^6B^-2 (http://web1.sph.emory.edu/PMEN/index.html). CC90 isolates were the main components of the OPNSP in our study. However, isolates belonging to this clone decreased in 2009–2011, and its substitutes, CC855 and CC3173, did not show higher β-lactam-antibiotic resistance. Therefore, this replacement cannot be explained by antibiotic overuse and remains undefined at present. Most of the CC855 and CC3173 isolates were identified as serotype 6A, which is not covered by the 7-valent pneumococcal conjugated vaccine (PCV7). The proportion of serotype 6A isolates in the OPNSP increased significantly (Table 4). This replacement may be attributable to immunization with PCV7, which was used after September 2008 in China. However, this rationale is not convincing because the vaccine immunization coverage was so low (less than 1%) that the spread of serotypes 19F and 23F was not restricted at all [26, 27].

The MIC_90_ and maximum MIC of penicillin in serogroup 6 *S. pneumoniae* isolates remained at 1 μg/ml and 2 μg/ml, respectively, from 2000 to 2010 in China (Table 2), whereas the MIC_90_ of serotypes 19 F and 23 F increased to 2 μg/ml and the maximum value was 8 μg/ml, and even reached 256 μg/ml [28, 29]. The present data also show the absence of salient resistant clones in serogroup 6 *S. pneumoniae*. They all may be under antibiotic selective pressure. Therefore, the CC/ST components of this group have not changed regularly, like serotypes 19 F and 23 F. This speculation corresponds to the decreasing frequency of serogroup 6 among all *S. pneumoniae* isolates over time [12]. The population biology of this serogroup should be monitored continuously in the future.

## Conclusions

In summary, this study clarifies the serotypes/subtypes and CCs/STs of group 6 *S. pneumoniae* present in China. Serotype “6E” was not identified in the traditional 6A isolates, and neither serotype “6 F” nor “6G” was assigned in our collection. Although the subtype 6B-II isolates were the main constituents of the OPNSP, their place was taken by serotype 6A isolates over time. The genetic constitution of the group 6 *S. pneumoniae* and its changes showed distinct regional characters. Unlike the serotype 19 F and 23 F isolates, no salient resistant clones were identified in serogroup 6 *S. pneumoniae*. Further long-term surveys of serogroup 6 *S. pneumoniae* are required to monitor the clonal prevalence and antimicrobial resistance in this important human pathogen.

## Authors’ original submitted files for images

Below are the links to the authors’ original submitted files for images. Authors’ original file for figure 1

## Abbreviations

PCR: Polymerase chain reaction
ST: Sequence type
MLST: Multilocus sequence typing
CC: Clonal complex
MDR: Multidrug resistant
MIC: Minimum inhibitory concentration.
