# Supplementary figures and images for: Population biology of 225 serogroup 6 Streptococcus pneumoniae isolates collected in China
Source: BMC Infect Dis. 2014 Aug 27;14:467. doi: 10.1186/1471-2334-14-467 (PMC4152590; doi:10.1186/1471-2334-14-467)

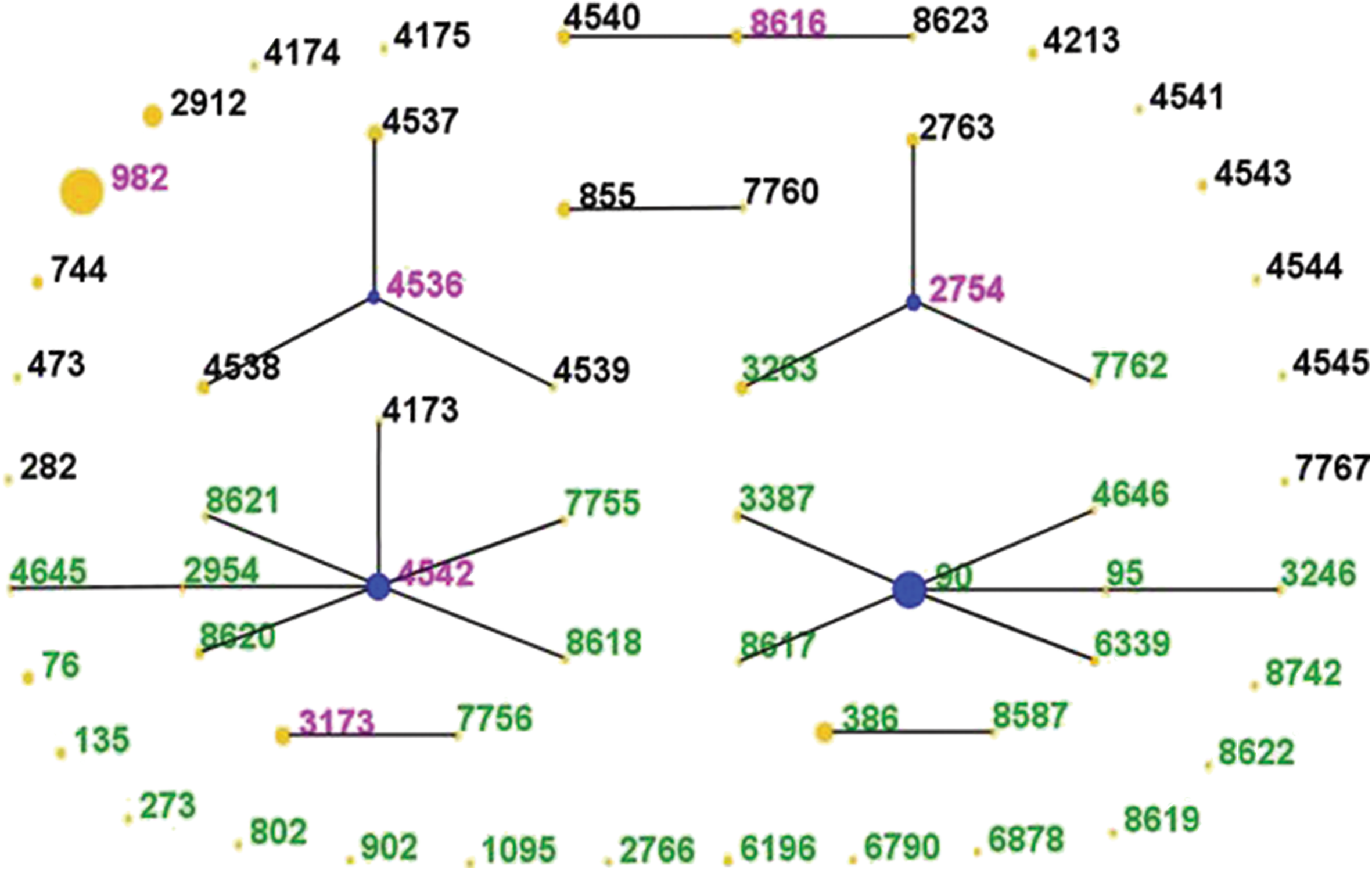

Supplement: Supplementary file 1 — Authors’ original file for figure 1 [file 12879_2014_3756_MOESM1_ESM.tif]
